# Supratentorial dural-based collision of cavernoma and meningioma: a case report

**DOI:** 10.1186/s41016-018-0128-5

**Published:** 2018-07-18

**Authors:** A. V. Dubovoy, V. M. Jafarov, E. I. Voronina

**Affiliations:** 1 FSBI “Federal Center of Neurosurgery”, Ministry of Health Care of the Russian Federation, Novosibirsk, Russian Federation 630087; 2 Ministry of Healthcare of the Russian Federation, Novosibirsk State Medical University, Novosibirsk, Russian Federation 630091

**Keywords:** Cavernous malformation, Cavernous hemangioma, Cavernous angioma, Meningioma, Convexity tumor, Collision tumor, Dural lesion

## Abstract

**Background:**

Collision tumor is a very rare case of cerebral lesion. Approximately 50 reports of intracranial collision tumors were described in the literature. We present a case of supratentorial dural-based convexity collision tumor radiologically mimicking cavernous malformation and composed of cavernous malformation and meningioma.

**Case presentation:**

The case presents a 63-year-old female having MR findings such as hemorraged supratentorial dural-based mass with “popcorn” signs and hemosiderin deposits. The patient underwent craniotomy and evacuation of the subdural hematoma with resection of the tumor mass. Histologically the lesion had signs of a cavernoma and meningioma. MRI in a year after surgery did not reveale residual tumor mass or recurrence.

**Conclusions:**

Collision tumor is a very rare case. Preoperative diagnosis of a dural lesion is difficult and challenging. A neuroradiological differential diagnosis of similar cases needs focused attention.

## Background

An intracranial collision tumor represents two coexisting, histologically distinct primary tumors in the same site without histological admixture or an intermediate cell population zone. Such tumors consist of components with different histogenesis and tumorogenic pathway representing a mosaic of two concurrent but independent tumors that have “collided” with each other [1]. Our report presents the case of a patient with supratentorial convexity dural-based lesion that have MR findings of cavernous malformation (CM) and composed both of CM and meningioma.

## Case presentation

**Fig. 1:**
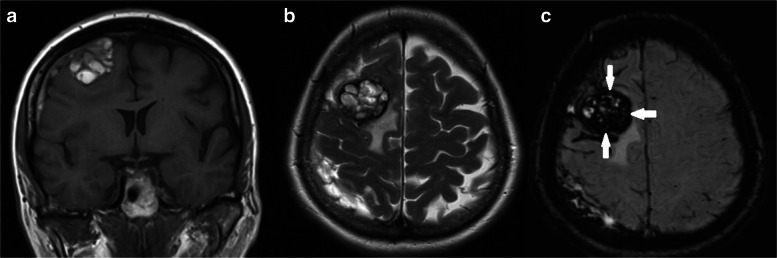
**a** Preoperative MRI. T1 weighted image, coronal plane. A mass sized 32 * 30 * 35 mm on the surface of the right frontal lobe, with heterogeneous iso- hyperintensive signal (lesion has «popcorn» sign), right chronic subdural hematoma with midline brainshift 3 mm. **b** Preoperative MRI. T2 weighted image, axial plane. This mass shows similar popcorn-shaped characteristics with perifocal edema. **c** Preoperative MRI. SWI sequence, axial plane. Ring of hypointensity around lesion (arrows)

A 63-year-old woman admitted in our clinic with complaints of intensive headaches in right frontal region and right orbit lasting for 1 month before. There was no history of vomiting, nausea, convulsions or unconsciousness, head trauma or early episodes of headaches. Physical and neurological examination was unremarkable. Magnetic resonance imaging showed a right chronic subdural hematoma with a slight midline brain shift and a tumor mass with a “dura-tail” and “popcorn signs” and hemosiderin deposits in the right frontal region (Fig. 1). Considering the cavernoma resection of this lesion was planned.

The patient underwent a right-side craniotomy using MRI-guided navigational assistance and neurophysiological monitoring. It was noted intraoperatively that the subdural hematoma had different stages of hemorrhages but predominantly chronic. There was no evidence of bone invasion or adjacent brain. The lesion was incapsulated and attached to the dura mater, capsule of mass had many veins. The tumor was dissected and removed away from the brain without bleeding. The resected mass had a brown-colored appearance with different size vessels in the cross section. A tight closure of the dura mater was performed using adjoining pericranium. Postoperatively the patient marked no neurological deficits.

**Fig. 2:**
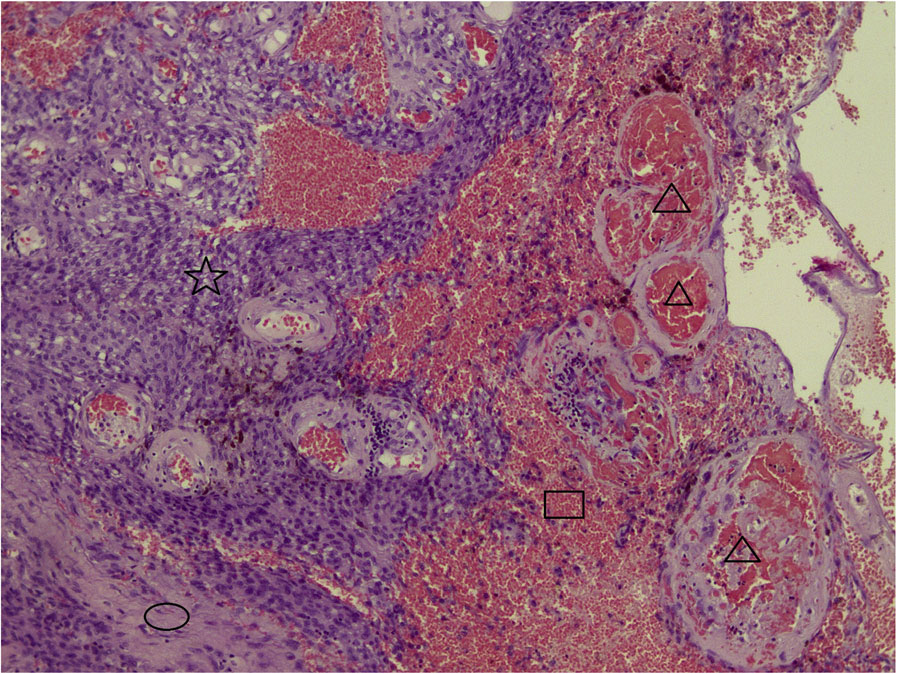
Collision of cavernoma and meningioma (H&E × 100). Different size thick- and thin-walled vascular channels (triangles), hemorrhages (rectangle), fibrous tissue (ellipse) and meningioma (star) on opposite site are presented

**Fig. 3:**
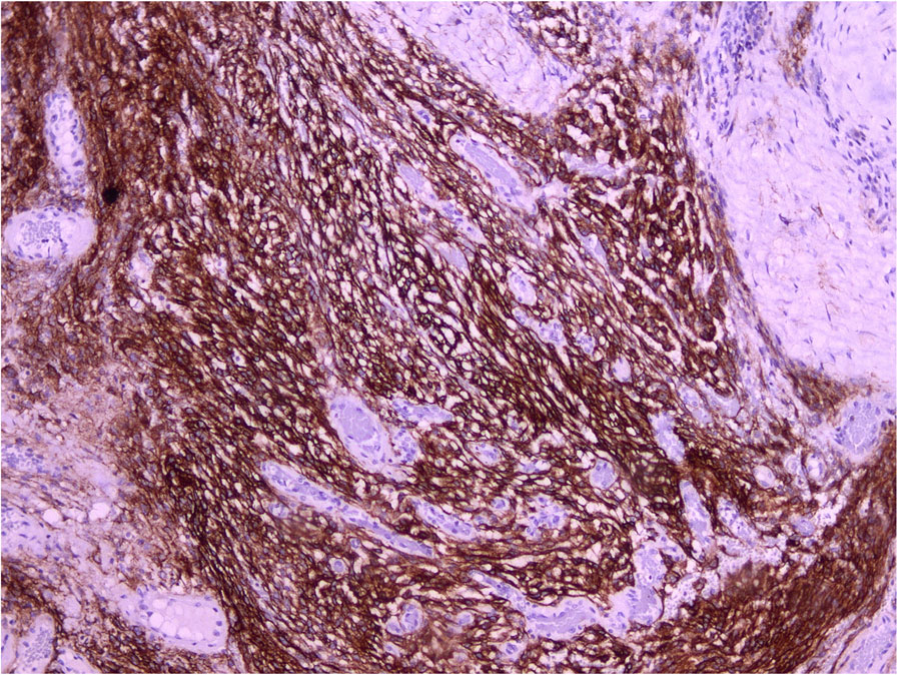
Cavernous malformation was confirmed by SMA. Another part of the tumor (× 100)

**Fig. 4:**
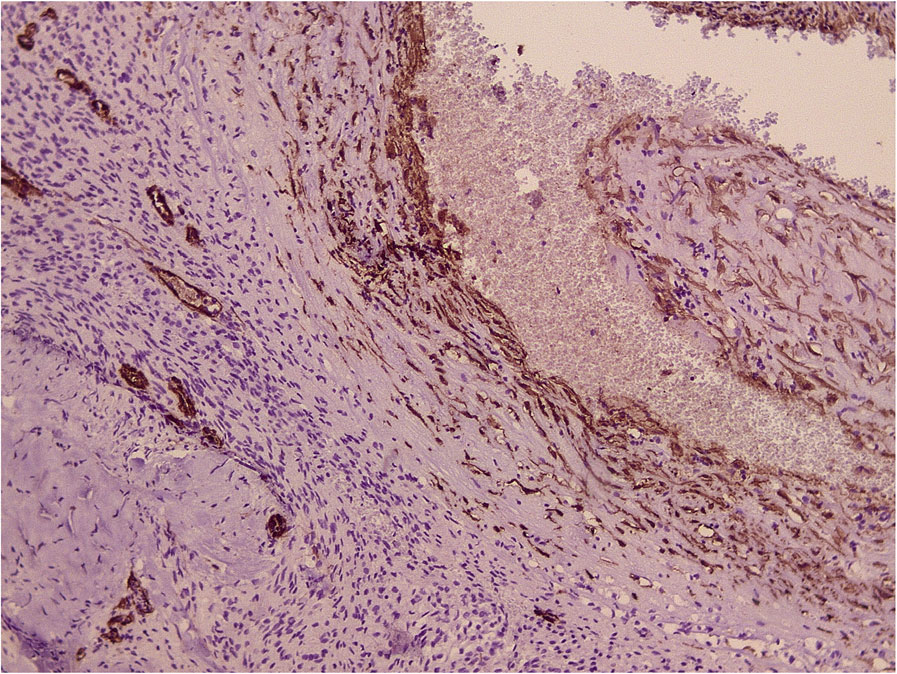
Meningioma (Grade I) was confirmed by EMA. Another part of the same tumor (× 100)

Histological examination revealed the following: macroscopically the removed tumor of round-oval shape, with dimensions up to 2.5 × 1.3 × 1 cm, brown color. Large multi-caliber vessels are defined on the section. Microscopically: among the focuses of the hemorrhages and fragments of the coarse-fibrous connective tissue are available the clusters of fulfilling different-sized vessels with unevenly dilated lumens, thickened and some places sclerotized walls, in some vessels there are erythrocyte-fibrin clots. Between the conglomerates of the vessels are determined the focuses of the tumor growth, consisting of meningotheliocyte-like cells, forming aggregations, the nuclei of the cells are light, moderately polymorphic, diffuse-focal interstitial lymphoid infiltration is defined, deposition of hemosiderin grains (Fig. 2). In the immunohistochemical study, fragments of formation regarded as meningiomas are positively stained with antibodies to epithelial membrane antigen (Fig. 3). A component regarded as CM was stained with antibodies to smooth-muscle actin – positive coloration on the walls of the blood vessels (Fig. 4). According to the morphological structure and immunomorphological characteristics, as well as on the basis of clinical data, removed tumor is regarded as the combination of meningioma (meningotheliomatous variant of the structure, Grade I) and vascular cavernous malformation.

**Fig. 5:**
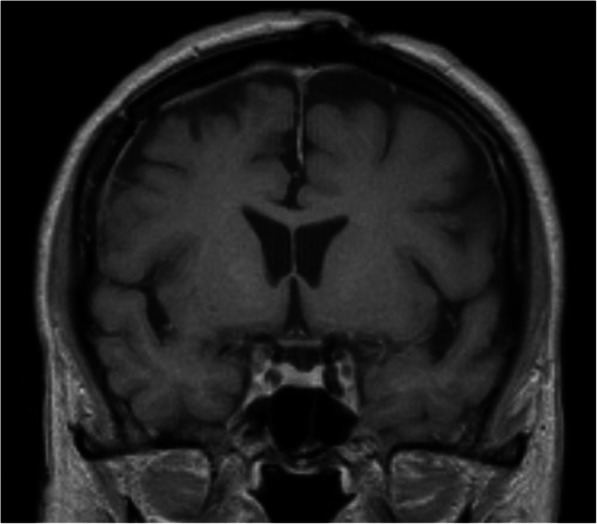
Postoperative MRI. T1 weighted image, coronal section. No residual or recurrent tumor mass

Postoperative course was uneventful, the patient felt well and her headaches resolved. She was discharged from hospital on the fifth day after the surgery. Follow-up course continued for more than 1 year and there was no recurrence of the preoperative symptoms. MRI examination showed no residual or recurrent tumor mass in the original tumor area (Fig. 5).

## Discussion

Approximately 50 reports of intracranial collision tumors were described in the literature. Most of them presented cases with collision tumors consisting of malignant astrocytoma (including glioblastoma) and meningioma. There are also reports of collision tumors consisting of two different cerebral metastases [1]. This report presents an unusual case of dural-based tumor mass mimicking CM and including both lesions – CM and meningioma.

Our preoperative diagnosis was cavernous malformation based on findings of brain MRI and included “popcorn signs”, the ring of hemosiderin on SWI, subdural hemorrhage on side of tumor mass. Clinical presentation was not specific. We were convinced that the tumor was a cavernoma until intraoperative visualization was done and proven with pathomorphology. Retrospectively this fact does not change surgical strategy – the similar tumor needs to be removed. Classical MRI features of pathologies can be misleading and decision of type of treatment in another case with hidden collision lesion may different. In this light more likely important that collision lesion had more risks of complication such as hemorrhage or hide high grade of tumor when lesion is small and asymptomatic.

Dural-based lesion in most cases is meningioma. Imaging characteristics of meningiomas include broad-based dural attachment, signal changes in the skull due to tumor infiltration, sharp demarcation between the tumor and the brain, mass effect on adjacent brain tissue and homogeneous contrast enhancement [2]. Cavernous hemangioma can closely resemble meningiomas on CT, MRI and angiography in terms of location, signal characteristics and enhancement pattern [3]. In MRI both lesions are iso- or hypointense on T1WI and iso- or hyperintense on T2WI with contrast enhancement. Dural tails, hyperostotic reactions and perifocal edema can also occur in both cases [3, 4]. The ring of hypointensity seen on MRI represents microhemorrhages and deposits of hemosiderin. SWI sequences are more sensitive in detecting microhemorrages and calcifications [4]. In our case the collision tumor had classical MRI features of cavernoma in one site. In another hand, Jadik et al. were the first who described the case of intraparenchymal meningioma mimicking CM [3].

Johnson et al. reported the neuroimaging differential diagnosis of dural lesions mimicking meningioma [5]. Some of these pathologies include solitary fibrous tumors, hemangiopericytomas, gliosarcomas, leiomyosarcomas, melanocytomas, metastatic neoplasms, tuberculomas, Hodgkin’s disease and plasmacytomas. In our opinion, cavernous malformation and collision tumor should be considered in differential diagnosis of dural-based lesion [3, 6, 7].

Some previous reports described dural-based CM that often mimic meningioma radiologically and vice versa [2–4, 6–9]. Vitantonio et al. reviewed 13 reports of dural-based CM mimicking meningioma [6]. Common features of it are elder ages (only in two cases there were 18-year-old and 15-year-old patients), headaches and parietal convexity localization. Some cases described severe facial pain similar trigeminal neuralgia due to dural irritation and CM eroding through the calvarium and presented initially as a soft scalp mass [8, 9]. The preoperative diagnostic of dural lesion in the light of the increasing cases of masquerading meningioma or cavernoma and also collision tumor needs focused attention. Unexpected findings in operating room or after histological confirming mistake of preoperative diagnosis and can change a treatment plan [4].

Weigel et al. reported the first case of collision tumor consisted of meningioma and cavernoma but ventricular localization [1]. In the reported case, collision tumor appeared after some surgical resections of a recurrent meningioma of left lateral ventricle and a cavernoma of caudal section of the aqueduct and dorsolateral part of the fourth ventricle. The authors noted that differential diagnosis between an angiomatous meningioma and the collision tumor is difficult and our results also prove it.

The correlation between cavernous hemangioma and meningioma remains unclear. The occurrence of these lesions could be coincidental. Weigel et al. based on ventricular localization of both tumors hypothesized that a possible explanation of the collision of the two different tumors may be a cerebrospinal fluid mediated migration of tumor cells [1]. Kilani et al. described some hypotheses based on radiation and traumas of head [10]. Our patient denied head history of trauma or cases of similar illness in the family history and also she did not get radiation. We agree with Kilani et al. that the relationship between the lesions suggests something more than a coincidental association.

## Conclusions

In summary, a collision tumor is a very rare case. Preoperative diagnosis of a dural lesion is difficult and challenging. A neuroradiological differential diagnosis of similar cases needs focused attention.

## Abbreviations

CM: Cavernous malformation
EMA: Epithelial membrane antigen
MRI: Magnetic resonance imaging
SMA: Smooth-muscle actin
SWI: Susceptibility weighted imaging
T1WI: T1 weighted image
T2WI: T2 weighted image

## Authors’ information

DA chief of the vascular division, member of EANS, ACNS, JV functional neurosurgeon, VE pathomorphologist.
